# Improving the Assessment of Neonatal Abstinence Syndrome (NAS)

**DOI:** 10.3390/children8080685

**Published:** 2021-08-09

**Authors:** Claire A. Chin Foo, Lynne M. Dansereau, Katheleen Hawes, Erica L. Oliveira, Barry M. Lester

**Affiliations:** 1 Brown Center for the Study of Children at Risk, Brown Alpert Medical School and Women and Infants Hospital, Providence, RI 02912, USA; LDansereau@wihri.org (L.M.D.); KHawes@Wihri.org (K.H.); EOliveira@Wihri.org (E.L.O.); 2 Department of Pediatrics, Brown Alpert Medical School and Women and Infants Hospital, Providence, RI 02908, USA; 3 Department of Psychiatry and Human Behavior, Brown Alpert Medical School, Providence, RI 02912, USA

**Keywords:** Neonatal Abstinence Syndrome (NAS), Neonatal Opioid Withdrawal Syndrome (NOWS), Finnegan Neonatal Abstinence Scoring System (FNASS), NICU Network Neurobehavioral Scale (NNNS)

## Abstract

Neonatal Abstinence Syndrome (NAS) is a public health problem of epidemic proportions. The Finnegan Neonatal Abstinence Scoring System (FNASS) is the tool most widely used to evaluate NAS. However, it is limited by its lack of interrater reliability and standardized approach. Surveys to evaluate the FNASS were distributed to nurses at the Women and Infants Hospital in Providence, RI, USA. Infants (*n* = 78) treated for NAS and born to methadone-maintained mothers were examined to compare items administered from the FNASS and the NICU Network Neurobehavioral Scale (NNNS). All nurses reported that the FNASS was somewhat to very subjective. More than half reported that it was somewhat to not accurate and a new scoring method is needed to accurately diagnose NAS. Correlations between FNASS items and NNNS items showed 9 of 32 (28.1%) correlations were strong (r_s_ > 0.5), 5 of 32 (15.6%) were moderate (0.3 < r_s_ < 0.5), and 10 of 32 (31.3%) were weak (0.1 < r_s_ < 0.3). Principal component factor analysis (PCA) of the NNNS explained more variance (35.1%) than PCA of NNNS and FNASS items combined (33.1%). The nursing survey supported the need for developing a more objective exam to assess NAS. NNNS exam items may be used to improve the evaluation of NAS.

## 1. Introduction

Opioid use during pregnancy has reached epidemic proportions with a 242% increase in the last 10 years [1]. Increases in maternal opioid use have been accompanied by a parallel increase in the drug withdrawal of opioid-exposed infants, known as Neonatal Abstinence Syndrome (NAS), or more recently, Neonatal Opioid Withdrawal Syndrome (NOWS) [2,3]. From 2000 to 2016, the incidence of NAS increased sevenfold from 1.2 to 8.8 per 1000 hospital births [2,3,4]. Hospital costs for NAS have increased more than sixfold since 2004, resulting in approximately $2 billion in excess costs primarily due to longer lengths of hospital stays [4].

The accurate diagnosis of NAS is critical because diagnosis of NAS typically leads to pharmacological as well as non-pharmacological treatment and an increased length of stay (LOS), which can result in additional exposure to opioids, affect the developing mother–infant relationship, and increase hospital costs. The most commonly used tool to evaluate the clinical manifestations in infants with NAS is the Finnegan Neonatal Abstinence Scoring System (FNASS), including modification, a 31-item list of signs and symptoms of three dimensions of withdrawal (Central Nervous System Disturbances; Metabolic, Vasomotor & Respiratory Disturbances; and Gastrointestinal Disturbances) based on record review, maternal reports, and direct observation [5,6,7]. However, limitations of the FNASS have been described, including a lack of interrater reliability and subsequent validation, as well as a lack of a standardized approach [7,8,9]. The current tools used to measure NAS, including the FNASS, have often been described as subjective and highly variable due to poor reliability, particularly for nurses who do not often care for babies with NAS [10,11].

The NICU Network Neurobehavioral Scale (NNNS) is a standardized, comprehensive evaluation that incorporates neurologic and behavioral measures and signs of stress [12]. The NNNS was originally developed as a research assessment for infants with prenatal cocaine and/or opioid exposure as part of the multisite longitudinal NIH Maternal Lifestyle Study. The NNNS has subsequently been used extensively across a broad spectrum of substance-exposed and other at-risk infant populations, is sensitive to NAS onset [13] and pharmacological treatment for NAS [14], as well as prenatal opiate exposure, and predicts developmental outcomes through age 4 ½ in this population [15]. Many of the symptoms of NAS that are scored on the FNASS are also scored on the NNNS. The NNNS is an objective, reliable, well-validated tool that is also used clinically with high-risk infants, including those with prenatal opioid exposure. An NNNS-based assessment may lead to improvements in the assessment and diagnosis of NAS and improve the management and treatment of this vulnerable population. In the current study, we conducted a survey about the FNASS with nurses who administer the exam, and we examined the relationship between FNASS and NNNS items measured in the same infants with NAS. The aim of the study was to determine if relations between Finnegan and NNNS items could lead to the development of an improved assessment of NAS.

## 2. Materials and Methods

Participants for the survey were 41 nurses who administer the FNASS in the Mother–Baby unit at the Women and Infants Hospital in Providence, Rhode Island. Because the survey was anonymous, we do not have information on the demographics of the participants, with the exception of the number of years they have been nurses and the number of years they have performed the FNASS. The survey we developed consisted of 15 questions that covered years of nursing experience, accuracy of the FNASS, subjectivity, and difficulty in scoring the items on the FNASS. The survey was developed by the first and third authors. Dr. Hawes is an advanced practice nurse with more than 13 years’ experience in nursing professional development and research in surveying nurse attitudes and perceptions related to the work environment and professional nursing practice. The survey was original and not tested previously. The survey included all the items on the FNASS to determine which ones the nurses found most subjective, which were most difficult to score, and which were most indicative of NAS (Supplementary Materials). Nurses completed the survey online or by depositing a paper copy into a survey-labeled sealed box in the nurses’ lounge. The study received IRB exemption due to the lack of personal health information or identifiable information about the participants.

The NNNS and FNASS comparison included 78 infants treated for NAS born to methadone-maintained mothers. Study details were explained, and informed consent was obtained in accordance with the institutional review board. Exclusion criteria were infant congenital anomalies or gestational age < 35 weeks, or maternal psychiatric problems that jeopardized informed consent. The FNASS was performed as part of standard care. FNASS training consists of studying the FNASS tool and instructions prior to observing experienced FNASS preceptors. Nurses observed their preceptors for three shifts (24–36 h total). During the training period the nurses score babies for interrater reliability with their preceptors. After the three-shift training period, nurses typically begin to score the FNASS on their own. The FNASS score was calculated by the nurse every 4 h from 2 h of age until 2 days after pharmacological treatment with morphine was discontinued. FNASS exams were administered throughout the infants’ length of stay and NNNS exams were administered prior to NAS treatment. Drug treatment began when 2 consecutive FNASS scores were ≥ 8. Drug treatment included oral neonatal morphine solution (0.4 mg/mL). The NNNS was performed before the FNASS 73% of the time with at least an hour in between exams. The NNNS is a 20–30 min well-validated exam that assesses active and passive muscle tone, primitive reflexes, movement, social behaviors (e.g., cuddling and soothability), attention to visual and auditory stimuli, and a checklist of stress signs organized by organ systems. The NNNS items are summarized into the following scales: Habituation, Attention, Arousal, Regulation, Handling, Quality of Movement, Excitability, Lethargy, Nonoptimal Reflexes, Asymmetric Reflexes, Hypertonicity, Hypotonicity, and Stress Abstinence. The NNNS was administered by trained NNNS examiners who were recertified every 6 months. The NNNS exam was matched to the FNASS that was closest in time to NNNS administration or the time of the last feed.

Maternal and infant characteristics were summarized using frequencies for categorical characteristics and means for continuous characteristics. FNASS and NNNS data were analyzed using SPSS software v24. [16]. Spearman rank-order correlation was used to measure the strength and direction of the association between FNASS and NNNS individual items. FNASS items and NNNS summary scale items were compared by organ system and items selected from the NNNS exam measured similar signs and symptoms as that of the FNASS. Items were strongly correlated at r_s_ > 0.5, moderately correlated at 0.3 < r_s_ < 0.5, and weakly correlated at 0.1 < r_s_ < 0.3. Principal component factor analysis (PCA) with varimax rotation was used to define the underlying structure among FNASS items and NNNS items that measure similar signs and symptoms with minimal information loss. We used a loading of > 0.40 to interpret the factor patterns.

## 3. Results

### 3.1. Nursing Survey

All nurses (100%) reported that the FNASS was “somewhat” to “very” subjective with more than half (56%) reporting that the FNASS was “somewhat” accurate to “not accurate at all”. The majority of the nurses (90%) thought that performing FNASS in the mother’s room compared to performing the FNASS in the nursery would impact the baby’s score. Half of the nurses (50%) thought that there were many nurse-to-nurse disagreements in the FNASS scoring system between nurses in the same unit of the hospital. When asked to describe the FNASS, 40% of nurses thought the FNASS was subjective and 57.6% of the nurses had a negative opinion of the FNASS in terms of length, complexity, and reliability. More than half of the nurses (51%) thought that there is a need for a new scoring method to diagnose NAS. We compared nurses’ years of performing the FNASS and years of nursing experience to the survey responses. Answers to the survey questions did not differ significantly by years of nursing experience or years of performing the FNASS (*p* > 0.05).

### 3.2. FNASS and NNNS

The majority of the infants’ mothers were white (91%), and 16.7% had not completed high school. The average gestational age of the infants was 38.4 weeks and 59% were male (Table 1).

Spearman rank-order correlations between items that measured similar constructs from both the FNASS and the NNNS summary scores were conducted to determine which items were most closely related (Table 2). Nine out of 32 (28.1%) items were strongly correlated (r_s_ > 0.5), 5 out of 32 (15.6%) were moderately correlated (0.3 < r_s_ < 0.5), and 10 out of 32 (31.3%) were weakly correlated (0.1 < r_s_ < 0.3).

PCA of the FNASS suggested a three-factor solution that explained 26% of the variance with eigenvalues of 2.2, 1.5, and 1.4. The first factor had four items: high pitched cry (0.75), sleep after feeding (0.50), Moro reflex (0.69) and nasal flaring (0.79). The second factor had three items: disturbed tremors (0.54), excoriations (−0.41), and fever (−0.47). The third factor had two items: undisturbed tremors (0.55) and sneezing (0.50).

PCA of NNNS items that measure similar signs and symptoms of the FNASS also suggested a three-factor solution that explained 35.1% of the variance with eigenvalues of 3.7, 1.5, and 1.4. The first factor had six items: predominant state (0.65), consolability (−0.68), peak of excitement (0.83), rapidity of build-up (0.68), irritability (0.73), and sucking (0.58). The second factor had four items: Moro reflex (0.46), tremulousness (0.48), general tone (0.62), and mottling (0.68). The third factor had three items: sweating (−0.49), nasal stuffiness (0.55), and loose or watery stools (−0.48).

**Figure 1:**
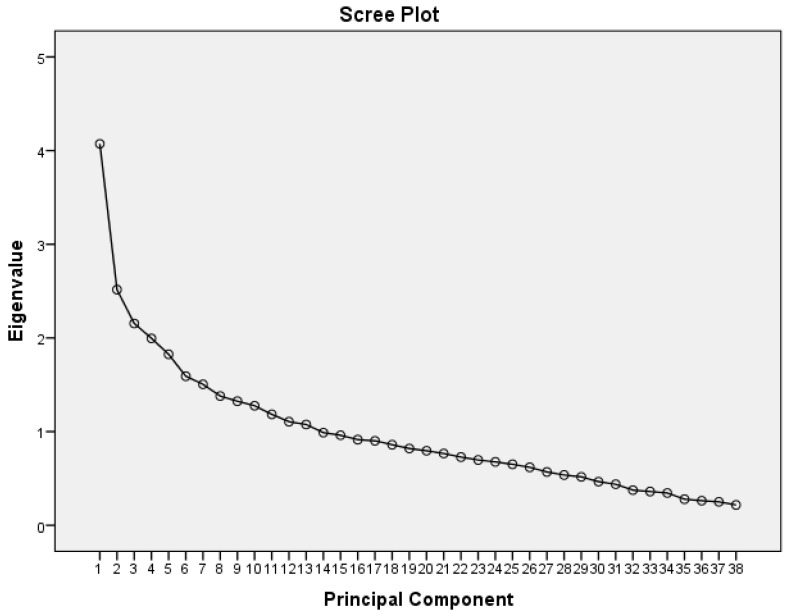
Scree plot from PCA of NNNS and FNASS.

PCA of the NNNS and FNASS items combined suggested a five-factor solution that explained 33.1% of the variance with eigenvalues of 4.1, 2.5, 2.2, 2.0, and 1.8 (Table 3, Figure 1). The first factor contained seven items all measured by the NNNS: predominant state, consolability, peak of excitement, rapidity of build-up, irritability, general tone, and sucking. The second factor had four items all measured by the FNASS: high pitched cry, sleep after feeding, Moro reflex and nasal flaring. The third factor had five items: high-pitched cry (NNNS), disturbed tremors (FNASS), excoriation (FNASS) and mottling (NNNS and FNASS). The fourth factor had six items: tremulousness (NNNS), sweating (FNASS and NNNS), nasal stuffiness (NNNS), and loose or watery stools (NNNS and FNASS). The fifth factor had four items: undisturbed tremors (FNASS), sneezing (NNNS and FNASS) and gastric issues (FNASS).

## 4. Discussion

We conducted a survey of nurses who care for opioid-exposed infants and solicited their evaluation of the FNASS to determine the need for an improved instrument to measure and diagnose NAS. Many nurses did not think the FNASS accurately diagnosed NAS; the majority described the FNASS as subjective and more than half believed a new scoring method is needed for a more precise evaluation. Another interesting finding was the nurses’ response to whether they thought that performing the FNASS in the mother’s room compared to the nursery would change the score. This suggests that the subjectivity of the exam is not only due to the items on the exam but also to the environment in which the FNASS is conducted. Hospital conditions vary in this respect with some exams conducted in the mother’s room, the nursery (where there are often other infants and additional distractions), or in a separate room such as a “feeding” room. When asked about scoring disagreements between nurses, half of the nurses reported there were many disagreements regarding the FNASS. Previous studies reported problems with reliability and validity of the FNASS that could compromise the clinical care and management of these infants [8]. Many inconsistencies in FNASS scoring have been reported and persisted even after training interventions at a six-month follow up in a previous study [9]. A 2014 study of nurses with 0–5 years nursing experience found that reliability was not established for single FNASS measures. [17]. In our study, the number of years of performing the FNASS and number of years of nursing experience did not influence survey responses. In summation, this survey supports previous reports from clinicians who use the FNASS suggesting at the very least the FNASS needs improvement or that a new exam might be warranted.

The correlations between individual items on the FNASS with similar items on the NNNS showed 24 of 32 statistically significant correlations with most of the correlations at the moderate or weak level. The majority of the correlations that were moderate or weakly correlated were CNS items and several CNS items were not correlated at all although they measure the same behavior. This suggests that the two instruments measure similar constructs but vary in their precision and reliability of measurement. It is also noteworthy that since the NNNS is based on the handling and direct observation of the infant and not chart review or maternal report, only Finnegan items measured in the same manner were used so that measurement error due to chart review or maternal report was not a factor.

Results of the PCA showed that the factor analysis of the NNNS explained more variance than the factor analysis of the FNASS. The PCA of the FNASS and NNNS items combined did not increase the percent of variance explained over that of the NNNS alone. Factor 1, the strongest factor from the NNNS and FNASS combined, included only NNNS items, six out of seven of which were related to CNS characteristics. This is noteworthy and suggests that the NNNS is more sensitive to CNS than other symptoms of NAS. The factor analysis findings also suggest that the NNNS is a structurally and psychometrically more robust instrument than the FNASS. In addition, the NNNS provides a comprehensive assessment of the neurobehavioral repertoire of the infant by measuring functional domains. For example, factor 1 from the PCA of the NNNS alone included six items: predominant state, consolability, peak of excitement, rapidity of buildup, irritability, and sucking. These are functional domains that are important beyond the assessment of NAS. These results suggest the possibility of developing an NNNS-based tool to better characterize NAS and provide additional information about the infant’s development.

The increase in NAS has shone the spotlight on diagnosis because treatment for NAS typically involves reintroduction of an opioid such as morphine. The infant then has to be weaned from morphine which can be a long, painful and expensive procedure. False-positive misdiagnosis of NAS can result in infants being unnecessarily treated with an opioid which means additional opioid exposure, longer hospital stays, increased costs, and mother–infant separation. False-negative misdiagnosis can result in infants not being treated when treatment is warranted. The danger here is that the infant is discharged and develops NAS at home, which would put the infant in danger and minimally require re-hospitalization.

The diagnosis of NAS is problematic because there is no “blood test” or other biomarkers, which forces us to rely on treating symptoms and clinical judgement. The FNASS has a long and storied history and originally was developed to determine the level of symptom severity that warranted pharmacological treatment [6]. Although the FNASS was not developed as a diagnostic tool, it has become the “gold standard” for the diagnosis of NAS as well. In fact, this distinction between the use of the FNASS as a diagnostic versus treatment tool is typically blurred. [18] This raises the question of whether we may need different metrics to diagnose NAS than to determine the threshold for the pharmacological treatment of NAS. In the absence of a biomarker, we have the mandate and the opportunity to revisit both the diagnosis and treatment of NAS based on the best current information that is available.

The concept of false positives and false negatives and measures of sensitivity and specificity do assume that there is a “gold standard.” Yet, in the case of the Finnegan, another unaccounted-for problem is the role of clinical judgement in its use. Is the exam meant to be a “clinical assist” device to provide “guidance” for clinical decision making or is it meant to provide quantifiable criteria to determine when to start (and maintain) opioid treatment? [7] In practice, studies of treatment variation suggest that clinical judgement is not used systematically across hospitals, which contributes to the variability in the treatment of NAS and means we have no standard of care [19].

Our findings here showed similarities between the FNASS and the NNNS. They measure similar constructs and have similar factor structures but differ in reliability and validity. This could be due to the precise and reliable descriptors in the NNNS, where examiners maintain at least 90% agreement on the items. Independent systematic reviews have concluded that interrater reliability and test–retest reliability for the NNNS is strong and that the NNNS has robust psychometric properties [20,21]. In addition, the NNNS is based solely on direct handling and observation of the infant, whereas the FNASS also includes information derived from maternal reports and the medical record. Items on the FNASS are inadequately defined with poor psychometric properties that leave room for subjectivity [9,17,22,23]. A number of attempts have been made to improve the FNASS by shortening the exam and trying to include only the most salient items by using statistical methods to determine the contribution of individual Finnegan items [24,25,26,27,28,29,30]. However, this begs the question: salient for what? For pharmacological treatment? For diagnosis of NAS, or for both? There may be a different set of items necessary for the diagnosis versus pharmacological treatment issue and there are undoubtedly items that are correlated with diagnosis and/or treatment but may not be necessary for either. Moreover, the issue of the role of clinical judgement still needs to be addressed.

There are a number of strategies that could be used to develop, in the absence of biomarkers, an assessment tool that improves the accuracy and reliability of the FNASS that is provided by the NNNS. The NNNS is based only on the direct handling and observation of the infant and provides detailed, carefully constructed behavioral descriptions with established reliability and validity assured by a rigorous training model. These modifications would dramatically reduce the subjectivity inherent in the FNASS. This is the first study, to our knowledge, to evaluate Finnegan and NNNS performance in the same group of infants. In addition, as we have seen in this study, comparisons of findings between the FNASS and the NNNS show that there are, as expected, statistically significant correlations between the FNASS and the NNNS; however, the psychometric characteristics of the NNNS, as indicated by the results of the factor analysis, suggest that the NNNS is a more robust exam than the FNASS. Notable is the findings that the strongest factor (factor 1) was composed only of NNNS items and that across factors, the items that comprised these factors were CNS-related, perhaps suggesting that items related to CNS activity are the most salient in describing NAS.

The NNNS exam has been used in hundreds of studies across a wide range of at-risk infants, including infants with NAS [13,14]. The NNNS is being increasingly used in clinical settings both nationally and internationally [31]. The exam is used in many hospitals as part of standard care to help with the management of specific populations, notably preterm infants and infants with prenatal substance exposure. It is also used clinically on a referral basis during the infant’s hospital stay and as part of discharge planning for the infant’s health care provider and early intervention referral. Moreover, the NNNS has long term predictive validity initially shown to predict IQ and problem behavior at age 4.5 in cocaine- and opioid-exposed infants [15]. Subsequent studies have confirmed the ability of the NNNS to forecast long-term developmental outcomes [32,33,34,35,36,37,38,39,40,41,42,43].

In conclusion, there seems to be widespread agreement that we need to improve our ability to assess and manage NAS and work toward developing a model of standard of care. In the absence of a biomarker, we must rely on symptoms, which is why trying to modify the Finnegan scale is a reasonable approach. We are suggesting a related but different approach which would not use the Finnegan items as they are but improve the measurement of those items that are determined to be most important by using an NNNS-based approach. The definitions, descriptions and scoring of items on the NNNS will likely improve the precision of measurement and enhance the psychometric properties of the tool. Moreover, the fact that the NNNS has been shown to predict long-term outcomes in infants with prenatal opioid exposure raises the possibility that an NNNS-based exam to diagnose NAS could ultimately be used to identify which infants with NAS are most likely to show long term developmental deficits [15]. The identification of these infants before hospital discharge could lead to the development of interventions to mitigate or prevent adverse developmental outcomes in infants with NAS. Critically, as suggested by Velez et al., the NNNS measures functional domains such as self-regulation that impact caregiver behavior and are important for later well-being [13]. Thus, an NNNS-based assessment of NAS could quantify specific strengths and weaknesses at the level of the individual infant that could identify functional neurobehavioral domains to target for intervention. Sharing this kind of information with caregivers could also strengthen the developing infant caregiver relationship and provide the bases for additional non-pharmacological care.

## Figures and Tables

**Table 1:** Maternal and infant characteristics at birth.

*n* (%) or Mean (SD)	*n* = 78
Maternal characteristics	
Race	
African American	2 (2.6%)
White	71 (91.0%)
Other	5 (6.4%)
Maternal age (yrs.)	29.1 (5.7)
Education, <12 years	13 (16.7%)
Parity	2.4 (1.3)
Infant characteristics	
Gender, male	46 (59.0%)
Gestational age (wk.)	38.4 (1.8)
Birth weight (g)	2968 (621)
Length (cm)	48.6 (2.8)
Head circumference (cm)	33.5 (2.9)
Apgar score, minute 1	7.5 (1.6)
Apgar score, minute 5	8.7 (0.6)

**Table 2:** Correlations between Finnegan and NNNS items.

Finnegan Item	NNNS Scale Item	Correlation Between Finnegan and NNNS Item	*p*-Value
**Central Nervous System Disturbances**			
Crying	High-pitched cry ***	0.827	<0.001
	Predominant state ***	0.732	<0.001
	Consolability	0.304	0.36
	Peak of excitement *	0.256	<0.001
	Rapidity of buildup **	0.452	<0.001
	Irritability **	0.334	<0.001
Reflexes	Moro reflex ***	0.705	<0.001
Tremors undisturbed—mild–severe	Tremulousness	0.089	0.41
	Low frequency/high amplitude ***	0.791	<0.001
	High frequency/low amplitude *	0.237	<0.001
Tremors disturbed—mild–severe	Tremulousness*	0.162	0.04
	Low frequency/high amplitude **	0.417	<0.001
	High frequency/low amplitude *	0.228	<0.001
Muscle tone	General tone	0.139	0.02
	Leg Resistance	0.074	0.19
	Popliteal angle *	0.165	<0.001
	Scarf sign *	0.192	<0.001
	Forearm resistance	0.089	0.12
	Truncal tone *	0.174	<0.001
Excoriation	Excoriations ***	0.749	<0.001
Myoclonic jerk	Myoclonic jerks	-	-
Generalized convulsions	Generalized seizures	-	-
**Metabolic, Vasomotor & Respiratory Disturbances**			
Sweating	Sweating	0.344	0.05
Frequent yawning	Yawning **	0.439	<0.001
Mottling	Mottling *	0.268	<0.001
	Skin color *	0.273	<0.001
Nasal stuffiness	Nasal Stuffiness ***	0.593	<0.001
Sneezing	Sneezing **	0.470	<0.001
Nasal flaring	Nasal flaring ***	0.677	<0.001
**Gastrointestinal Disturbances**			
Excessive sucking	Sucking	0.027	0.75
Stools	Stools ***	0.554	<0.001
Regurgitation	Spit-up ***	0.647	<0.001

* Weak correlation (0.1 < r_s_ < 0.3). ** Moderate correlation (0.3 < r_s_ < 0.5). *** Strong correlation (r_s_ > 0.5).

**Table 3:** Factor Loadings on FNASS and NNNS items.

	Component				
	Factor 1	Factor 2	Factor 3	Factor 4	Factor 5
Eigenvalue	4.1	2.5	2.2	2.0	1.8
% Variance	10.7	6.6	5.7	5.3	4.8
**Central Nervous System Disturbances**					
High-pitched Crying (FNASS)		0.744			
High-pitched cry (NNNS)			−0.403		
First predominant State (NNNS)	0.611				
Consolability with intervention (NNNS)	−0.582				
Peak of excitement (NNNS)	0.780				
Rapidity of build-up (NNNS)	0.751				
Irritability (NNNS)	0.816				
Sleep after feeding (FNASS)		0.562			
Moro Reflex (FNASS)		0.625			
Tremors: Undisturbed (FNASS)					0.586
Tremors: Disturbed (FNASS)			0.405		
Tremulousness (NNNS)				0.422	
General tone—Predominant tone (NNNS)	0.522				
Excoriation (FNASS)			−0.538		
**Metabolic/Vasomotor/Respiratory Disturbances**					
Sweating (FNASS)				−0.530	
Sweating (NNNS)				−0.552	
Mottling (FNASS)			0.446		
Mottling (NNNS)			0.622		
Nasal stuffiness (NNNS)				0.404	
Sneezing (FNASS)					0.601
Sneezing (NNNS)					0.465
Nasal Flaring (FNASS)		0.727			
**Gastrointestinal Disturbances**					
Sucking (NNNS)	0.489				
Stools (FNASS)				−0.522	
Gastric Issues (FNASS)					0.516
Loose stools, watery stools (NNNS)				−0.514	

## Data Availability

Data sharing not applicable.

## Supplementary Materials

The following are available online at https://www.mdpi.com/article/10.3390/children8080685/s1, supplementary: Nursing opinion on the finnegan score survey.
