# Supplementary material for: Improving the Assessment of Neonatal Abstinence Syndrome (NAS)
Source: Children (Basel). 2021 Aug 9;8(8):685. doi: 10.3390/children8080685 (PMC8394483; doi:10.3390/children8080685)
Supplement: Supplementary file 1 [file children-08-00685-s001.zip › children-1277469-supplementary.pdf]

Nursing Opinion On The Finnegan Score Survey

Welcome to My Survey

Informed Consent:

Thank you for participating in this survey. Your feedback is important. Please answer the following questions as honestly as possible. These questions concern the Finnegan scoring system and your opinion concerning the Finnegan. As you are aware, the Finnegan Neonatal Abstinence Scoring System (FNASS) is currently the gold standard for determining babies' level of NAS.

The purpose of this survey is to help determine a general opinion of the FNASS.

I do not anticipate that taking this survey will contain any risk or inconvenience to you. Furthermore, your participation is strictly voluntary and you may withdraw your participation at any time without penalty.

All information collected will be used only for my research and will be kept confidential. There will be no connection to you specifically in the results or in future publication of the results. Once the study is completed, I would be happy to share the results with you if you desire. In the meantime, if you have any questions please ask or contact:

Claire Chin Foo, Research Assistant at Women & Infants Hospital, phone: 401-274-1122 ext 48984

Or

Barry M. Lester, PhD, Director of the Center for the Study of Children at Risk, phone: 401-274-1122

Additionally, if you have any concerns about your treatment as a participant in this study, please call:

The Director of IRB Administration at (401) 453-7677.

Although the chairperson may ask your name, all complaints are kept in confidence.

By submitting the survey, you are verifying that you have read the explanation of the study, and that you agree to participate. You also understand that your participation in this study is strictly voluntary.

1. How long have you been working as a nurse?

☐ 0 – 3 years ☐ 4 – 6 years ☐ 7 – 9 years ☐ 10 – 12 years

☐ 13 – 15 years ☐ 16 – 19 years ☐ Over 20 years

2. How long have you been doing FNASS evaluations?

☐ 0 – 3 years ☐ 4 – 6 years ☐ 7 – 9 years ☐ 10 – 12 years

☐ 13 – 15 years ☐ 16 – 19 years ☐ Over 20 years

3. What training method did you have to perform the FNASS?

☐ Net Learning (videos)
☐ Classes
☐ Certified Finnegan Training
☐ Nurse to nurse
☐ None

4. After the training you received, how prepared did you feel to perform the FNASS?

☐ Not at all prepared ☐ Somewhat prepared ☐ Prepared ☐ Very prepared ☐ N/A

5. How accurate do you think the FNASS is at capturing babies' withdrawal symptoms?

☐ Not accurate ☐ Somewhat accurate ☐ Accurate ☐ Very accurate

6. How subjective do you find the FNASS? (subjective meaning that some items are broadly defined or not well defined)

☐ Not at all subjective ☐ Somewhat subjective ☐ Subjective ☐ Very subjective

7. Which environmental factors do you think impact the babies' symptoms and therefore the FNAS score they receive? *Check all that apply*

☐ Light
☐ Noise
☐ Commotion in the nursery (busy, many parents, many babies)
☐ Mother's availability (spend time with baby)
☐ Nursery staff availability

8. Which of the following interventions have you tried to reduce these environmental factors? *Check all that apply*

☐ Reduce lighting
☐ Reduce noise
☐ Use soothing music
☐ Encourage mom to be available for her baby
☐ Limit number of people in the nursery and in the room

9. Do you think that performing the FNASS in the mother's room compared to the nursery can affect the FNAS score?

☐ Yes
☐ No

10. **What items do you think are the most difficult to score?** *Check all that apply*

Central Nervous System Disturbances

- ☐ Crying: Excessive High Pitched
- ☐ Crying: Cont. High Pitched
- ☐ Sleeps < 1 Hr After Feeding
- ☐ Sleeps < 2 Hr After Feeding
- ☐ Sleeps < 3 Hr After Feeding
- ☐ Hyperactive Moro Reflex
- ☐ Markedly Hyperactive Moro Relex
- ☐ Mild Tremors: Disturbed
- ☐ Mod-Severe Tremors: Disturbed
- ☐ Mild Tremors: Undisturbed
- ☐ Mod-Severe Tremors: Undisturbed
- ☐ Increased Muscle Tone
- ☐ Excoriation (Specific Area)
- ☐ Myoclonic Jerk
- ☐ Generalized Convulsions

Metabolic, Vasomotor & Respiratory Disturbances

- ☐ Sweating
- ☐ Fever < 101 (37.2-38.3c)
- ☐ Fever > 101 (38.4c)
- ☐ Frequent Yawning (>3)
- ☐ Mottling
- ☐ Nasal Stuffiness
- ☐ Sneezing (>3)
- ☐ Nasal Flaring
- ☐ Respiratory Rate (>60/Min)
- ☐ Respiratory Rate (>60/Min w/Rtx)

Gastrointestional Disturbances

- ☐ Excessive Sucking
- ☐ Poor Feeding
- ☐ Regurgitation
- ☐ Projectile Vomiting
- ☐ Loose Stools
- ☐ Watery Stools

11. **What items do you think are the best indicators in diagnosing NAS?** *Check all that apply*

Central Nervous System Disturbances

- ☐ Crying: Excessive High Pitched
- ☐ Crying: Cont. High Pitched
- ☐ Sleeps < 1 Hr After Feeding
- ☐ Sleeps < 2 Hr After Feeding
- ☐ Sleeps < 3 Hr After Feeding
- ☐ Hyperactive Moro Reflex
- ☐ Markedly Hyperactive Moro Relex
- ☐ Mild Tremors: Disturbed
- ☐ Mod-Severe Tremors: Disturbed
- ☐ Mild Tremors: Undisturbed
- ☐ Mod-Severe Tremors: Undisturbed
- ☐ Increased Muscle Tone
- ☐ Excoriation (Specific Area)
- ☐ Myoclonic Jerk
- ☐ Generalized Convulsions

Metabolic, Vasomotor & Respiratory Disturbances

- ☐ Sweating
- ☐ Fever < 101 (37.2-38.3c)
- ☐ Fever > 101 (38.4c)
- ☐ Frequent Yawning (>3)
- ☐ Mottling
- ☐ Nasal Stuffiness
- ☐ Sneezing (>3)
- ☐ Nasal Flaring
- ☐ Respiratory Rate (>60/Min)
- ☐ Respiratory Rate (>60/Min w/Rtx)

Gastrointestional Disturbances

- ☐ Excessive Sucking
- ☐ Poor Feeding
- ☐ Regurgitation
- ☐ Projectile Vomiting
- ☐ Loose Stools
- ☐ Watery Stools

12. **Do you think that there are many nurse to nurse disagreements in FNAS scoring between nurses in the same unit of the hospital?**

- ☐ Yes
- ☐ No

**13. Do you think that there are many nurse to nurse disagreements in FNAS scoring between nurses in different units of the hospital?**	
☐ Yes<br>☐ No	
**14. How would you describe the FNAS?** *Check all that apply*	
☐ Accurate	☐ Subjective
☐ Easy	☐ Complex
☐ Short (items and time doing the exam)	☐ Long (items and time doing the exam)
☐ Reliable way to measure NAS	☐ Not reliable
**15. Do you think a new scoring method is necessary to diagnose NAS?**	
☐ Yes<br>☐ No	

Thank you very much for you time!
